# General practitioners’ barriers and facilitators towards new provider-initiated HIV testing strategies: a qualitative study

**DOI:** 10.1177/0956462416652274

**Published:** 2016-05-20

**Authors:** Ivo K Joore, Sanne LC van Roosmalen, Jan EAM van Bergen, Nynke van Dijk

**Affiliations:** 1 Department of General Practice/Family Medicine, Division of Clinical Methods and Public Health, Academic Medical Center, Amsterdam, The Netherlands; 2 Amsterdam Institute for Global Health and Development (AIGHD), Amsterdam, The Netherlands; 3 STI AIDS Netherlands (Soa Aids Nederland), Amsterdam, Netherlands; 4 Epidemiology & Surveillance Unit, Centre for Infectious Disease Control, National Institute of Public Health and the Environment (RIVM), Bilthoven, The Netherlands

**Keywords:** Qualitative research, HIV, general practice, primary health care, guidelines, epidemiology

## Abstract

European guidelines recommend offering an HIV test to individuals who display HIV indicator conditions (ICs). UK guidelines recommend performing a ‘routine offer of HIV testing’ in primary care where HIV prevalence exceeds 2 in 1000. Implementation of new provider-initiated HIV testing strategies in general practice is limited, while the numbers of undiagnosed and late for care HIV patients remain high. We have explored Dutch general practitioners’ barriers to and facilitators of both strategies. We combined semi-structured in-depth interviews with focus groups. Nine general practitioners – key informants of sexually transmitted infection/HIV prevention and control – were selected for the interviews. Additionally, we organised focus groups with a broad sample of general practitioners (n = 81). Framework analysis was used to analyse the data. Various barriers were found, related to (1) the content of the guidelines (testing the right group and competing priorities in general practice), (2) their organisational implementation (lack of time, unclear when to repeat the HIV test and overlong list of ICs) and (3) the patient population (creating fear among patients, stigmatising them and fear regarding financial costs). Multiple general practitioners stated that performing a sexual risk assessment of patients is important before applying either strategy. Also, they recommended implementing the IC-guided approach only in high-prevalence areas and combining HIV tests with other laboratory blood tests. General practitioners tend to cling to old patterns of risk-based testing. Promoting awareness of HIV testing and educating general practitioners about the benefits of new provider-initiated HIV testing strategies is important for the actual uptake of HIV testing.

## Introduction

An estimated total of 22,100 individuals are living with HIV in the Netherlands, 12% of whom are undiagnosed.^1^ The annual number of new HIV diagnoses is approximately 1000 new diagnoses in recent years.^1^ In 2014, 44% of newly diagnosed patients presented late for care (CD4 count < 350 cells/mm^3^ or with an AIDS-defining event regardless of CD4 count).^1^ Early diagnosis and treatment of HIV is an important strategy in fighting the HIV epidemic, with public and individual health benefits.^2–4^ These findings that early treatment enhances life-expectancy and reduces HIV transmission demonstrate the importance of early case finding.

General practitioners (GPs) are the primary point of access to healthcare, also for sexually transmitted infection (STI) care. The majority of healthcare for people with STIs is provided by GPs and STI clinics.^5,6^ Around 70% of STI consultations in the Netherlands take place at the general practice, and more than 30% of the HIV patients in care are diagnosed in this setting.^1,7,8^

In most national guidelines, provider-initiated HIV testing among high-risk groups for HIV is recommended.^9^ However, this approach has certain intrinsic limitations towards the implementation.^7,10–12^ GPs and patients may find it difficult to discuss detailed questions about HIV testing and sexual behaviour.^11^ Also, GPs may not always follow the guidelines.^7^ Overall, this strategy alone is not sufficient in reducing the number of undiagnosed and late for care HIV patients. Other new provider-initiated HIV testing strategies need to be explored.

Two new HIV testing strategies have been issued recently.^13–16^ The European Centre for Disease Prevention and Control recommends offering a HIV test to individuals who display HIV indicator conditions (ICs), which are defined as: (a) conditions which are AIDS-defining among people living with HIV; (b) conditions associated with an undiagnosed HIV with prevalence of >0.1%; and (c) conditions in which missing a diagnosis of HIV infection may have significant adverse implications for the individual’s clinical management.^9,13^ Another new HIV testing strategy, from the National Institute of Health and Care Excellence (NICE) in the UK, recommends a ‘routine offer of HIV testing’ to all 15–59-year-olds in primary care settings where HIV prevalence exceeds 2 in 1000. This strategy is operationalised in two ways: offering an HIV test (a) when registering new patients and (b) to anyone who has a laboratory blood test, regardless of the reason.^14,15^ In 2015, UNAIDS concluded that a more focused public health response is needed for fighting the HIV epidemic using better data to map high HIV prevalence areas and populations at higher risk of HIV, while combining the most cost-effective strategies.^17,18^

Both of the new provider-initiated strategies have been developed to make HIV testing a more routine behaviour for doctor and patient alike, and thereby avoid complex conversations about sexual risk behaviour.^13–15^ They could therefore help to normalise the use of the HIV test.^5,6^ In order to assess successful implementation of both new provider-initiated HIV testing strategies, we described GPs’ barriers to and facilitators of both strategies in early case finding.

## Methods

We used a qualitative approach to answer our questions, combining semi-structured in-depth interviews with focus groups of GPs.

### Recruitment and sampling

Between January and February 2014, nine GPs working in urban or rural Dutch practices were approached as key informants for a semi-structured in-depth interview (Table 1). For these interviews, GPs with special interest in STI/HIV were selected. The goal of the interviews was to gain a broad view on the various related components of the subject in order to frame the focus groups. They were performed at their general practice by a trained researcher (IKJ). We aimed to explore the opinions of GPs with different levels of experience of STI/HIV prevention and control. To do this, we organised six mixed focus groups. We expected that the large number of GPs, located in and around Amsterdam, in both interviews and focus groups would provide us with a broad range of views about new provider-initiated HIV testing strategies. Also, GPs during the focus groups could challenge each other to refine their insights of new HIV testing strategies. In April 2014, 81 GPs took part in these six focus groups following a multilevel educational intervention at the Department of General Practice of the Academic Medical Center (AMC) in Amsterdam, the Netherlands (Table 1). The multilevel educational intervention offered training in STI/HIV testing skills to GPs. After the intervention, GPs should be able to diagnose and treat the majority of STI/HIV themselves. The first training day provided the GPs with a high level of knowledge about STI/HIV prevention and control. During the six months before the focus groups (second training day), they were able to reflect what they have learned in daily practice and what barriers and facilitators related to the new provider-initiated HIV testing they encountered, which enhanced the possibility to discuss real-life barriers and opportunities instead of theoretical ones. All the participating GPs were also trainers of GP registrars.

**Table 1.** Characteristics of participating GPs in semi-structured in-depth interviews and focus groups.

	Semi-structured in-depth interviews	Focus groups
Total N	9	81
Gender		
Male	44.4%	51.9%
Median age (IQR)	46 (43.5–56.5)	51 (46.5–57.0)
Professional experience		
>10 years	77.8%	81.5%
Nationality		
Dutch	100%	98.8%
Number of HIV patients in practice		
<5 patients	22.2%	63.0%
5–10 patients	33.3%	22.2%
10–25 patients	0	6.2%
>25 patients	44.4%	6.2%
Unknown	0	2.5%
Location of practice		
City	77.8%	76.5%

IQR: Interquartile range.

Socio-demographic information and informed consent were obtained from all participants in the interviews and focus groups.

The semi-structured interviews started with an introduction about the study and stated that more HIV testing is needed in general practice to reduce the number of undiagnosed and late for care HIV patients. Interviews lasted between 60 and 90 min. After questions about GPs experience with HIV patients in their practice, we asked about their opinion of new HIV testing strategies with help of open-ended questions, for example, ‘What is your opinion about new HIV testing strategies?’ and ‘How would you implement these strategies in your practice?’.^9,13–15^

The focus groups started with a reflection on the preceding educational programme and a statement concerning the relevance of the study and the need for more provider-initiated HIV testing and were guided by a prewritten topic guide and slides. Participants were asked about their opinion of new provider-initiated HIV testing strategies published as guidelines.^9,13–15^ The focus groups lasted between 60 and 75 min and were facilitated by an independent moderator and observer. All moderators and observers were staff researchers in the Department of General Practice of the AMC in Amsterdam.

### Data analysis

All the interviews and focus groups were audio taped and transcribed verbatim. Additionally, the observers’ notes were used for analysis. Two researchers analysed the text independently and discussed the findings with the principal investigator (NvD). One of the researchers (IKJ) is involved in several studies about HIV testing in primary care and has a background in STI/HIV prevention and control. The other (SCR) is experienced in qualitative research, but not in STI/HIV prevention and control. Data immersion was achieved through a repetitive process of open coding to familiarise us with the data and to determine the categories of GPs barriers to and facilitators of both strategies. After analysis, the two researchers held debriefing discussions until consensus was reached on newly emerging barriers and facilitators. With each new transcript, newly emerging barriers and facilitators were added until data saturation was achieved. A third researcher (NvD) resolved differences over the ranking of categories through discussion and consensus. In the end, relevant barriers and facilitators were combined to make up a framework. All transcripts were analysed using MAXQDA software.

## Results

### Participants

In all, nine GPs were recruited for the semi-structured in-depth interviews and 81 for the focus groups. The characteristics of these 90 participants are described in Table 1. Based on their statements and on the number of HIV patients in their practices, we concluded that urban GPs with more experience of STI/HIV prevention and control had a more open attitude towards both provider-initiated strategies than their rural counterparts. GPs with more interest in STI/HIV had a more general positive attitude towards these new strategies and better understood the need for expanded testing.

GPs experienced various barriers related to the content, organisation and patient-related aspects of both strategies. They also mentioned several facilitators, which might be useful to GPs in implementing either strategy.

### Content-related barriers

Participants doubted if either strategy tests the right target group. A high proportion felt resistance towards the high number of HIV tests the two strategies would entail, with high percentages of negative results, even though both have been shown to be cost-effective.

With this strategy we have to do a lot of HIV tests. I understand that you have to do this with certain ICs, like persistent leukopenia or thrombocytopenia and recurrent pneumonia, but other diseases like herpes zoster are too prevalent. (Focus group: IC-guided testing)

Many GPs were not in favour of offering an HIV test to all patients from a high-prevalence area or with ICs, the respective approaches recommended by the two strategies. Participants felt they would end up testing too many people.

You are not testing the target group. Do you want to test every 40-year-old Dutch person in a high-prevalence area? I think this is not efficient, because you focusing upon the wrong target group. (Interview: the routine offer of testing)

With regard to IC-guided testing, participants doubted the definitions and scientific basis of some of the ICs, as they were not clearly presented in the guidelines.

To which grade of cervix dysplasia should I offer the HIV test? (Focus group: IC-guided testing)

Herpes zoster, is that adequately supported by evidence? (Focus group: IC-guided testing)

Also, participants mentioned that other issues in general practice were more important than HIV.

Interviewer: Offering an HIV test to all newly registered patients at a general practice?

GPs answer: Patients would be better off being tested for cholesterol and other health problems. (Focus group: the routine offer of testing)

### Organisational barriers

Participating GPs mentioned that discussing an HIV test would take time, and they were afraid that offering one could not be done during the consultation in which the patient first presented.

You need extra time to discuss the HIV test. For example, if an elderly person comes to you with a completely different problem like diabetes. (Interview: the routine offer of testing)

Participants also suggested that offering HIV tests to new patients registering at their practice was inappropriate, as they have yet to establish a relationship with these individuals.

Testing for HIV is indirectly linked to somebody’s personal lifestyle. Offering an HIV test is not a good idea when there is no connection with your patient. (Focus group: the routine offer of testing)

Moreover, participants felt unsure if or when to repeat the HIV test under either strategy. Or when to repeat it if a patient might have an acute HIV infection, when there is a window period after infection during which they will not test positive. During that time you can test HIV-negative even though you are infected, although newer tests have shortened these periods compared with those described in our guidelines.^9,19^

The problem is that you are not finished. You will have to repeat the HIV test every three months. (Focus group: the routine offer of testing)

This means we have to repeat the HIV test every eight weeks. (Interview: IC-guided testing)

In the case of IC-guided testing, participating GPs thought the list of ICs was too long and not applicable in primary care. Also, GPs stated that they would not immediately think of HIV when faced with certain of the ICs mentioned in the guidelines.

GPs will not remember all these ICs. (Focus group: IC-guided testing)

Symptoms like fever and feeling sick are observed frequently. If someone came back from a holiday in Ghana with, for example, you wouldn’t think of HIV first. You might think of malaria. GPs don’t immediately connect these symptoms with HIV. (Interview: IC-guided testing)

### Patient-related barriers

Some participants were concerned that patients would refuse the HIV test because of the cost. GP consultations in the Netherlands are covered by mandatory health insurance, but all insured persons also have to pay a compulsory excess themselves.^20^ This worry was specifically acute amongst those participants working in multicultural and socioeconomically deprived areas.

I notice in my practice that a lot of people don’t want an STI test because they have to pay for it themselves. This is not a good development. (Focus group: the routine offer of testing)

Participants were further concerned about judging their patients’ sexual behaviour and raising stigma if they discussed an HIV test when ICs were diagnosed.

I do not think it’s appropriate to offer an HIV test to a 15-year old with symptoms of mononucleosis-like illness. I’m not going to do that (Focus group: IC-guided testing)

Some participants stated that IC-guided testing might create unnecessary fear among patients.

A lot of fear is created by this strategy, because you need to discuss HIV with the patient. (Focus group: IC-guided testing)

### Facilitators

In respect of both strategies, participants stated that collecting more information about somebody’s sexual behaviour or adding age restrictions to frequently diagnosed ICs could help with patient selection.

Knowing if somebody is at risk for HIV is important. I would not immediately offer an HIV test to a 55-year-old woman with a laboratory blood test. (Interview: the routine offer of testing)

If you added an age restriction to the indicator-guided strategy, it would probably be more cost-effective. (Focus group: IC-guided testing)

With indicator-guided testing, one member of a focus group suggested that implementing this in a high-prevalence area might be more beneficial.

I think this is much easier in a high-prevalence area, where HIV is more prevalent. (Focus group: IC-guided testing)

Participants were more likely to test for HIV when there is a clear link with immunodeficiency, because the relationship between HIV and the immunodeficiency-related diseases is clear.

Recurrent pneumonia has a clear association with immunodeficiency. It will be easier for GPs to test for HIV. (Focus group: IC-guided testing)

To facilitate remembering to test, one participant advised integrating ICs into other relevant national guidelines for GPs.

Testing for HIV in patients with seborrheic dermatitis has to be implemented in GPs’ national guidelines for skin diseases. (Interview: IC-guided testing)

With respect to the routine offer of testing, general health checks involve multiple tests on a person who does not feel ill with the purpose of finding disease early, preventing it from developing or providing reassurance.^21^ Some of the participants recommended general health checks, combining the HIV test with other laboratory blood tests, as a possible way to implement the routine offer of testing approach.

Maybe you should combine an HIV test with a cholesterol test. Combining different relevant laboratory blood tests might work. (Interview: the routine offer of testing)

## Discussion

The guiding principle behind both provider-initiated strategies is that avoiding a risk assessment bypasses some of the barriers GPs encounter and so could help to accelerate HIV testing.^5,6^ In our study, various barriers were found to both strategies. These related to (1) their content, (2) their organisational implementation and (3) the patient population. Many GPs stated that performing a sexual risk assessment of patients is important before applying either strategy, implying that they tend to cling to old patterns of risk-based testing.

Routine offer of HIV testing in pregnant women using the ‘opt-out strategy’ has been implemented successfully in the Netherlands since 2004.^22^ In 2012, almost 99.8% of pregnant women participated in the HIV screening programme. The success of this routine offer of testing strategy indicates that avoiding sexual risk assessment may be one of the reasons for the high participation rates. However, the participants in our study remained in favour of collecting additional information about a person’s sexual risk profile were either new strategy to be implemented, even though that would be at odds with its guiding principle in both cases. GPs need to become aware that, by avoiding a risk assessment, the two provider-initiated strategies could help normalise HIV testing.

A Dutch survey investigating how often GPs discuss the topic of sexual behaviour with patients in relation to health risks reported that only 5% had done so within the past five years. By contrast, 83% of patients felt that GPs were entitled to discuss their sexual behaviour.^23^ Our data confirm that GPs’ own personal barriers, such as believing their patients would feel uncomfortable discussing HIV, still exist.^11^ A qualitative UK study reported that the public finds the offer of testing for HIV upon GP registration acceptable in high-prevalence areas.^24^ However, GPs in our study suggested that offering a test to new patients registering at their practice would be inappropriate, as they have yet to establish a relationship with these individuals. Patient and GP perspectives of the new HIV testing strategies may well be at variance. Considering both points of view in future interventions to implement either strategy might facilitate that process.

In our study, GPs mentioned that the list of ICs was too long.^13^ Electronic clinical reminder systems (CRs) in the electronic medical record could help GPs to remember ICs. A US study involving the implementation of a CR effectively increased HIV testing among primary care patients not previously tested, whereas education and practice feedback alone did not.^25^ However, the use of CRs in primary care remains fraught with obstacles: healthcare providers do not use them, are resistant to them, are concerned that their systems will become overloaded with them, find them onerous and believe that they interfere with their practice.^26–28^

Some of the GPs in our study recommended general health checks, combining HIV testing with other laboratory blood tests. Comparable high-risk groups are described for HIV and for hepatitis B and C.^9,29^ In a UK cross-sectional study of new immigrants, laboratory blood tests in primary care were used to detect multiple infections. This study showed that the routine offer of HIV testing was feasible in the primary care context and acceptability was high.^30^ However, more insight is needed to see if this approach would be relevant, feasible and cost-effective in primary care settings. Moreover, it remains a matter of debate whether general health checks, screening programs, should be a part of GP-practice, which is primarily aimed at individual patient care.

The strengths of this study are that we interviewed GPs working in urban and rural settings and with different levels of interest and experience in STI/HIV prevention and control, representing many different views about both strategies.

This study has several limitations. GPs that took part in these six focus groups following a multifaceted educational intervention or GPs with more interest in STI/HIV could have been biased for being more open and positive towards new HIV testing strategies. Remarkably, GPs with more interest in STI/HIV prevention and control did not have better understanding of the advantages of the new provider-initiated HIV testing strategies.

The median age of GPs being interviewed was quite high, which may influenced the high number of barriers we found. Some of the GPs may not be up-to-date with the current evidence and still frame HIV as an exceptional disease with coexisting barriers around HIV testing.^11^

Multiple GPs in our study had less than five HIV patients in their practice and may not be that experienced or interested with STI/HIV prevention and control. As a consequence, barriers towards both new strategies could have received more attention than facilitators. We aimed to prevent this by informing participants extensively on both strategies, to make sure all facilitators and possible barriers would receive adequate attention. Also, as all focus groups were on the same day given the organisation of the training, analysis of the data between groups to steer following meetings was not possible. Through the high number of focus groups, however, we are quite certain that all relevant aspects were discussed and data saturation was achieved.

At the time of our research, no information was available on where high-prevalence areas for HIV, defined according to the criteria of the NICE guidelines, in the Netherlands are located. A limitation of this study could be that many of the opinions concerning the ‘routine offer of HIV testing’ strategy came from GPs not active in such an area. However, all participants were informed that that strategy was indicated specifically for high-prevalence areas. Nevertheless, our sample did include GPs from large cities, including Amsterdam, which have a higher than average population of patients with HIV.^1,29^

This study has addressed many GPs’ barriers to and facilitators of both strategies, which can be integrated into future interventions. GPs never mentioned that these two strategies could help normalising the HIV test and reduce stigma which is a very important message for implementation of both strategies. How the HIV test is offered in both strategies, is important if patients are to agree to be tested and helps to overcome the barrier lack of time.^12,31^ To operationalise both new HIV testing strategies, the GP can more easily say to patients: ‘with these ICs an HIV test is recommended’, without the need to perform a sexual risk assessment.

GPs were concerned that patients would refuse the HIV test because of the costs. To offer this HIV test for free or for a cheaper rate to their patient could be a solution and discussion of this topic on policy level is warranted.

Not all ICs recommended in European guidelines are diagnosed by GPs. We advise researchers to determine a specific list with ICs commonly seen in primary care.^13^ Also, insight in a country’s high-prevalence areas, according to the UK guidelines, is important for developing the routine offer of HIV testing in these areas.^14,15^

Promoting awareness of HIV testing and educating GPs about the benefits of new provider-initiated HIV testing strategies is important for the actual uptake of HIV testing.

## Availability of data and materials

Providing data to other parties requires additional consent from the participants. Data can be accessed by authorized persons at the department of General Practice of the AMC-UvA in line with the privacy and ethical regulations.
